# Applying the Plan-Do-Study-Act cycle in medical education to refine an antibiotics therapy active learning session

**DOI:** 10.1186/s12909-021-02886-3

**Published:** 2021-08-30

**Authors:** Stacey Rose, Richard Hamill, Andrew Caruso, Nital P. Appelbaum

**Affiliations:** 1 Department of Internal Medicine, Section of Infectious Diseases, Baylor College of Medicine, One Baylor Plaza, Suite M220.15, Houston, TX 77030 USA; 2 Department of Internal Medicine, Baylor College of Medicine, Houston, TX USA; 3 Division of Evaluation, Assessment, Research, Department of Surgery, Baylor College of Medicine, Houston, TX USA

**Keywords:** Active learning, Antibiotics therapy, Medical education, Quality improvement

## Abstract

**Background:**

Active learning improves learner engagement and knowledge retention. The application of continuous quality improvement methodologies, such as the Plan-Do-Study-Act (PDSA) framework, may be useful for optimizing medical education, including active learning sessions. We aimed to enhance student satisfaction and achievement of learning outcomes by applying the PDSA framework to an antibiotic utilization curriculum for medical students.

**Methods:**

Guided by the Plan-Do-Study-Act framework, between February 2017 and July 2019, we developed, implemented, and revised an active learning session for medical students, focused on appropriate utilization of antibiotics during their Internal Medicine clerkship.

**Results:**

Across twelve sessions, 367 students (83.4%) completed the post-evaluation survey. Although baseline ratings were high (97% of respondents enjoyed the “active learning” format), constructive comments informed iterative improvements to the session, such as modifying session timing, handouts and organization of the gaming component. Intervention 3, the last improvement cycle, resulted in more favorable ratings for the active learning format (*p* = 0.015) improvement in understanding antibiotics and their clinical application (*p* = 0.001) compared to Baseline ratings.

**Conclusions:**

This intervention suggests that active learning, with regular incorporation of student feedback vis-à-vis a PDSA cycle, was effective in achieving high student engagement in an Internal Medicine core clerkship session on antibiotic therapy. Iterative interventions based on student feedback, such as providing an antibiotic reference table and answer choices for each case, further improved student receptivity and perceived educational value. The study findings have potential implications for medical education and suggest that the application of the PDSA cycle can optimize active learning pedagogies and outcomes.

**Supplementary Information:**

The online version contains supplementary material available at 10.1186/s12909-021-02886-3.

## Background

Active learning and continuous quality improvement are critical strategies when designing and refining medical school curricula [1, 2]. Active learning pedagogies “change the teacher–student relationship to a learner–learner relationship,” allowing learners to both engage in and reflect upon the learning experience [1]. Active learning modalities, including games, are known to increase knowledge retention [3–5]. Medical educators, both at our institution and nationwide, are deliberately re-evaluating their curricula to incorporate active learning instructional methodology [1, 6]. In parallel, medical educators also strive for continuous improvement of educational sessions, including those using active learning [2]. Quality improvement paradigms, including the Plan-Do-Study-Act (PDSA) cycle, are typically applied to clinical or organizational realms, but may also be relevant for informing a trajectory for improving the receptivity and efficacy of an educational intervention [7]. The Model for Improvement [8], which is the foundation for the iterative PDSA approach, focuses on three core questions: 1) What are we trying to accomplish, 2) How will we know that a change is an improvement, and 3) What change can we make that will result in improvement?

Continuous quality improvement (CQI) in curriculum development is recognized by the United States Liaison Committee for Medical Education (Element 1.1) as a critical standard in medical education [9]. However, the methodical integration of improvement cycles such as PDSA are often lacking in practice, largely due to the high resource requirements of frequent programmatic assessments and data review [10]. Despite the broad application of PDSA cycles in quality improvement paradigms, and the emphasis on CQI in curriculum development, there remains a paucity of data regarding the usefulness of applying the PDSA cycle to improve medical education sessions [11, 12].

For this study, we chose to focus on improving education concerning appropriate utilization of antibiotics - an increasingly important topic in medical education, particularly given the public health implications of antibiotic stewardship [13]. In an effort to enhance student engagement and retention of core concepts in infectious diseases, we developed and implemented an active learning session for medical students on the Internal Medicine core clerkship. The session aimed to improve student understanding of antibiotic mechanisms of action, side effects, and appropriate utilization for commonly encountered infections. We then applied the PDSA model and developed interventions to improve subsequent sessions in an iterative fashion, based on quantitative and qualitative feedback from participating students.

In this paper, we describe the development and implementation of an active learning session for medical students, focused on appropriate utilization of antibiotics. We also describe how the PDSA cycle informed curricular improvement over time, as measured by student-reported satisfaction and achievement of learning outcomes.

## Methods

### Active learning innovation

Between February 2017 and July 2019, we developed and implemented an active learning session for medical students on the Internal Medicine clerkship at Baylor College of Medicine. Sessions occurred once per clerkship rotation and are part of the formal didactics for the course; attendance is mandatory.

The learning objectives for our session, entitled “Antibiotic Therapy,” were for students to 1) differentiate the major antibiotic classes including mechanism of action and major side effects and 2) demonstrate knowledge of appropriate antibiotic utilization for commonly encountered infections (such as urinary tract infections, pneumonia, skin / tissue infections). Two infectious disease physicians [SR, RH] facilitated each offering of the session, with each session having approximately forty medical students in attendance.

The original instructional design for the session included two components, both of which incorporated gaming: 1) completion of an antibiotic chart and 2) case-based learning. In the first segment, students received an antibiotic chart, including drug classes and examples, mechanism of action, spectrum of disease(s) targeted by the antibiotic class, and side effects. The chart was intentionally incomplete, however, and students were encouraged to work in small groups (5–10 students) to fill in the blanks using online reference materials. The first group to complete the chart received candy, and then aided the faculty in presenting key concepts to the other students through large group discussion. A completed version of the chart is available in Additional file 1.

In the second segment, the students reviewed seven clinical cases to highlight common infectious conditions and appropriate antibiotic utilization. The format for this segment was modeled after an active learning demonstration at IDWeek 2016 [14]; however the cases and answers were developed entirely by the faculty instructors. Each case was read aloud, then students worked in small groups (5–10 students) to list the relevant “bugs and drugs” on an index card and then placed the card in a paper bag. The first group to place a card in the bag with the correct “bugs and drugs” was provided with candy, and was asked to describe their answers and rationale to the rest of the students. The facilitators helped to highlight important teaching points or provide additional commentary, including correcting any inaccuracies in the student presentations. The total time allotted for the session – including both the antibiotic chart completion and case discussions – was one hour. If time did not permit discussion of all cases, students received copies of the cases and correct answers following the session. The cases, answers and facilitators guide are available in Additional file 2.

Following each session, the faculty instructors reviewed qualitative comments and ratings from a post-session evaluation survey to inform modifications to future sessions as appropriate based on student feedback. Faculty reviewed all comments, and identified comments that provided constructive feedback (i.e. recommended a change to the session). The faculty prioritized implementation of recommended changes based on frequency (such as if several students had similar recommendations) and feasibility. Once a change was implemented, the evaluation data and student comments were re-examined in order to assess how students perceived the change. The process thus involved continuous improvement, similar to a PDSA cycle model (Table 1). In addition to session changes based on student feedback, the faculty also periodically reviewed and updated the material in the antibiotic chart based on the available literature.

**Table 1:** ACT Phase of the PDSA framework: Session modifications based on student feedback

Phase	Rationale	Student Qualitative Feedback Received During this Phase	Date of first implementation
Baseline Phase: Provided students an incomplete antibiotic chart to complete during the session as a small group activity. Also completed a small group case activity on “bugs and drugs” identification.	Engage students for the majority of session time on active learning activities.	*“I enjoyed the interactive nature of the lecture; however, I think it was a little too hectic to learn as much as I would have liked.”* *“The table filling part was basically only for people who’d already done ID consult. I want to learn those things, I just don’t think that was a good use of our lecture time.”* *“Send out Abx chart before the lecture.”* *“Only suggestion would be to move to a classroom better for groups.”* *“The bag thing could change, but was a lot of fun.”*	2/17/2017
Intervention 1: Provided students with pre-populated antibiotic chart (including medications, mechanisms, adverse effects, etc.)	Students did not like the antibiotic chart completion activity and wanted more time for case discussions	*“I would use something other than [bag] to submit answers. Other than that, good!”* *“Thanks for putting together the chart- super helpful.”*	7/27/2018
Intervention 2: For the case-based learning, students could hold up their hands once they had a list of “bugs and drugs” for the case (rather than submit their answers by placing a card into a paper bag)	Students indicated that submitting answers using the paper bag was impractical based on the layout of the classroom (lecture hall with theater seating)	*“Would like to get answers. Chart very helpful.”* *“Put answer choices on same page as vignettes.”*	1/15/2019
Intervention 3: For the case-based learning, students were provided with a list of possible answer choices following each case (rather than providing a separate packet with potential answer choices); this minimized the need for students to consult multiple resources during the session	Students requested better organization of the materials used for the session (i.e. combining the packet of cases with the packet of answer choices)	*“It was perfect! And very helpful. The only thing is that I wish we have more time/went faster.”* *“Antibiotic chart is very helpful! Consider sending digital version we can reference on wards.”* *“Really great review! I wish we had more time. I learned new things as well.”* *“Love the concise, helpful table at the end.”*	5/17/2019

### Post-session evaluation survey

Following each session, students completed a brief evaluation which asked them to rate session quality (enjoyment of the “active learning” format, appropriateness of the content, and whether the lecture improved their understanding of antibiotic utilization) using a five-point Likert scale (1-strongly agree; 5-strongly disagree). The evaluation also included an open response question for “other comments or suggestions” to facilitate qualitative feedback.

Likert items were analyzed through SPSS Version 26 (Armonk, NY) via descriptive statistics (count/percentage—“Strongly agree” and “Agree” statements were summed to indicate “Satisfaction”) and nonparametric group comparison tests (Kruskal-Wallis Test) were conducted between the four intervention phases. The four intervention phases were identified as Baseline (six sessions between 02/17/17–02/23/18), Intervention 1 (two sessions between 7/27/18–10/19/18), Intervention 2 (two sessions between 01/15/19–3/22/19), and Intervention 3 (two sessions between 05/17/19–07/26/19). Follow up pairwise comparison tests further identified significant group differences with adjusted Bonferroni corrections for multiple comparisons. Qualitative data from the open response items were themed by each of the four intervention phases; if any part of a respondent’s comment had a constructive element, then it was automatically categorized as a constructive comment. The Institutional Review Board for Human Subject Research for Baylor College of Medicine and Affiliated Hospitals approved our research protocol through expedited procedures. An earlier version of this work has been published online (https://www.sciencegate.app/keyword/197386).

## Results

### Quantitative data

**Fig. 1:**
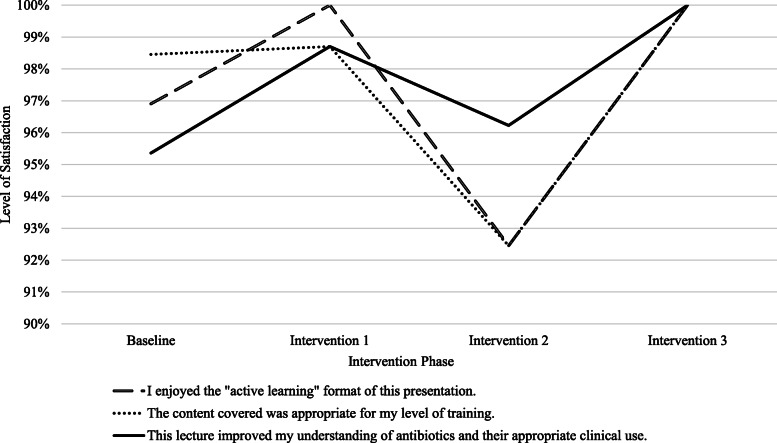
Students’ Level of Satisfaction with Antibiotics Session. Note: Fig. 1 reflects the percentage of students who strongly agreed or agreed (SA/A) with each statement across the four intervention phases. Statistical comparisons across intervention phases can be found in Table 2. Across all twelve sessions (sample size n=367): 96% (n=354) of students SA/A that they enjoyed the “active learning” format of this presentation; 97% (n=356) of students SA/A that the content was appropriate for their level of training; 96% (n=352) of students SA/A that the lecture improved their understanding of antibiotics and their appropriate clinical use

The number of students within each phase varied (Baseline, 6 sessions, *n* = 221; Intervention 1, 2 sessions, *n* = 94; Intervention 2, 2 sessions, *n* = 63; Intervention 3, 2 sessions, *n* = 62). Across all twelve sessions, 367 of 440 students (83.4% response rate) completed the post-evaluation survey. Students’ level of agreement with the three Likert-items varied across the four intervention phases (Fig. 1). Nearly all students were satisfied or very satisfied with the active learning format, appropriateness of content-level for trainees, and improved understanding of antibiotics and their appropriate clinical use after Intervention 3, the last round of PDSA improvement efforts. Enjoyment with the active learning format and perceived improvement in understanding antibiotics and their appropriate clinical application had at least one significant difference between intervention phases (Table 2). Intervention 3 resulted in more favorable ratings for the active learning format, improvement in understanding antibiotics, and their appropriate clinical application compared to Baseline ratings (Table 2).

**Table 2:** Pairwise Comparisons for Intervention Phases

Evaluation Item	Kruskal-Wallis Test Statistic	Significant Pairwise Comparisons
I enjoyed the “active learning” format of this presentation.	H (3)= 10.52, p = 0.015	Intervention 3 (Mean Rank = 160) vs. Baseline (Mean Rank = 196) (*p* = 0.043)
The content covered was appropriate for my level of training.	H (3)= 3.44, *p* = 0.328	–
This lecture improved my understanding of antibiotics and their appropriate clinical use.	H (3)=15.98, p = 0.001	Intervention 1 (Mean Rank = 170) vs. Baseline (Mean Rank = 200) (*p* = 0.038) Intervention 3 (Mean Rank = 158) vs. Baseline (Mean Rank = 200) (p = 0.012)

Note: Pairwise comparisons corrected for multiple comparisons. We only included significant pairwise comparisons. Lower mean ranks indicate higher agreement on item

### Qualitative data

When prompted with “*Other comments or suggestions*”, 228 respondents provided a text comment. There were 48 constructive comments during the Baseline phase compared to 20 during Intervention 1, 11 during Intervention 2, and 5 during Intervention 3. We analyzed general themes through coding efforts; multiple themes within a single comment were broken out. The most frequent constructive themes at Baseline were (1) students did not enjoy the charting activity (2) the session was too fast paced and (3) students wanted to cover more content and/or more cases during the session. The most frequent constructive themes at Intervention 1 were (1) the lecture needed to be longer (2) desire for more groups with fewer members, while Intervention 2’s constructive themes were (1) better content organization (2) desire for more groups with fewer members, and (3) more time allocated to group discussion. At Intervention 3, the only constructive theme was that the session needed to be longer: “*I wish the session was longer so we could cover all the cases*” and “*It was perfect! And very helpful. The only thing is that I wish we have more time/went faster.*”

## Discussion

We developed and implemented an active learning session for Internal Medicine clerkship students on antibiotic utilization, with deliberate incorporation of student feedback to modify future sessions. In our study, the use of a PDSA cycle, with iterative modifications based on qualitative student feedback, led to more favorable student ratings in terms of the session format, self-assessed understanding of antibiotics, and their appropriate clinical application. Narrative comments also improved with associated changes in the constructive themes. Even at baseline, student satisfaction with the “active learning” format of the presentation was high (97% of respondents were strongly agreed or agreed that they enjoyed the “active learning” format of the session). Our findings are consistent with the growing literature on gaming in medical education, which suggests high levels of enjoyment and engagement [15], particularly for sessions designed to include opportunities for accomplishment, discovery and bonding [5]– all of which were incorporated into the instructional design for this session.

Despite the high baseline satisfaction, we introduced intentional modifications to the session format based on student feedback. We removed the first part of the session (student completion of an antibiotic chart) in order to have more time for the clinical case discussions, altered the means by which groups could submit their answers (raising their hands rather than placing index cards in a paper bag), and improved organization of the handouts. Following each intervention, student evaluations provided evidence whether interventions improved the sessions and to identify additional areas for improvement. Of note, Intervention 1 was purposefully implemented after six sessions of observation. Considering this was a novel educational session, we felt it was important to ensure robust baseline analysis in the “Study” phase before reengaging in the “Act” phase of the PDSA cycle.

The quantitative data showed statistically significant improvements over time for both learner enjoyment of the session format (baseline vs. Intervention 3; *p* = 0.043), and learner-rated understanding of antibiotics and their appropriate clinical use (baseline vs. Intervention 3; *p* = 0.012). Moreover, the content of the qualitative comments evolved over time, such that the only constructive theme identified in the final phase was a request for the session to be longer. Both the quantitative and qualitative data suggest that the iterative modifications, driven by student feedback, were successful in addressing student concerns and improving the quality and impact of the session.

Of note, the quantitative data did show a decline during the Intervention 2 phase; data during this phase represents a cohort of students who were on their first clinical rotation, and may thus have felt less comfortable with the session format and content as compared to students who are further along in their clinical training. For example, one student commented, “*Some of the content was a little above my level of training; however this could be b/c of my lacking in understanding*.” Alternatively, the decline during the Intervention 2 phase may belie limitations in the reliance on self-report as part of the applied PDSA framework. Correlation with objective measures of knowledge acquisition would be useful in parallel, but such data was not part of the study design. Interestingly, the qualitative data from the Intervention 2 group indicated a high level of satisfaction and reflected the successful incorporation of the previous session improvements. As one student commented: “*Thanks for putting together the chart- super helpful*” – directly reflecting the impact of Intervention 1 (removal of the chart completion exercise).

Not all suggested modifications to the sessions were feasible for implementation. For example, several student comments centered on the time allotted for the session, requesting more time for case discussions; however, the instructors were not able to lengthen the session time allotted due to the limitations of the Internal Medicine clerkship didactic structure. Overall, the strengths of this study include the high response rate and multimodal data collection (quantitative and qualitative), which allowed the faculty to implement and assess the impact of iterative changes to the session format based on student feedback.

Some study limitations include that each student participated in the session once (as part of the Internal Medicine clerkship), therefore the evaluation data reflects different cohorts of students. Thus, the change in results over time may be due to respondent variability rather than based on interventions. Additionally, the survey asked students to rate their enjoyment of the “active learning” format, though there were modifications to the format over time, resulting from the implementation of constructive feedback. For example, at Baseline, students were actively completing the antibiotic table, but this was later provided as a pre-populated resource. Such changes may have impacted student perceptions. Finally, assessment of the impact of the session in achieving the learning objectives relied on learner self-ratings, rather than an independent knowledge assessment. Notably, data from the Internal Medicine National Board of Medical Examiners assessment – an objective assessment taken by all students on the core Internal Medicine clerkship at our institution - showed improvement in student performance between 2017 and 2019 in the area of “Health Maintenance, Pharmacotherapy, Intervention & Management” (mean performance increased from 0.2 to 0.5 above the SD for the national comparator cohort); however, this is a very indirect measurement of learning outcomes and cannot be attributed to a single active learning session on antibiotic utilization.

Future directions for this work may include the incorporation of additional sources of data, such as a standardized assessment of knowledge and application of core concepts in antibiotic utilization, and expanding the time for the session as suggested by learners in multiple phases of the study.

## Conclusions

The PDSA cycle, well established in the quality improvement literature, appears relevant for medical education and curricular improvement efforts. Systematic use of student feedback to drive iterative instructional modifications, with subsequent analysis of the impact of each intervention, appears advantageous for improving student receptivity and perceived educational value of an active learning session. The concept of continuous quality improvement, or CQI, is often employed only at the level of programmatic assessments; however, our findings suggest there is value in using “CQI” practices to drive curricular improvement at the level of an individual didactic session. We successfully demonstrated the application of the PDSA cycle for purposeful improvement of a specific educational session on antibiotic therapeutics. Given the increasing importance of training in antibiotic stewardship for medical trainees, the study findings have potential implications for medical education, and suggest that the use of active learning, combined with regular incorporation of student feedback via a PDSA cycle, are useful tools for curriculum develop in antibiotic stewardship.

## Supplementary Information

**Additional file 1.** Antibiotic Chart.

**Additional file 2.** Antimicrobial Therapeutics Cases: Facilitator’s Guide.

## Data Availability

The data analyzed in the current study are available from the corresponding author on reasonable request.

## Abbreviations

PDSA: Plan-Do-Study-Act
CQI: Continuous Quality Improvement

## References

[1] Graffam B (2007). Active learning in medical education: strategies for beginning implementation. Med Teach.

[2] Blouin D, Tekian A (2018). Accreditation of medical education programs: moving from student outcomes to continuous quality improvement measures. Acad Med.

[3] Felszeghy S, Pasonen-Seppänen S, Koskela A, Nieminen P, Härkönen K, Paldanius KMA, Gabbouj S, Ketola K, Hiltunen M, Lundin M, Haapaniemi T, Sointu E, Bauman EB, Gilbert GE, Morton D, Mahonen A (2019). Using online game-based platforms to improve student performance and engagement in histology teaching. BMC Med Educ.

[4] Schuller MC, DaRosa DA, Crandall ML (2015). Using just-in-time teaching and peer instruction in a residency program’s core curriculum: enhancing satisfaction, engagement, and retention. Acad Med.

[5] Singhal S, Hough J, Cripps D. Twelve tips for incorporating gamification into medical education. MedEdPublish. 2019;8(3). 10.15694/mep.2019.000216.1. 10.15694/mep.2019.000216.1 PMC10712530 38089323

[6] McCoy L, Pettit RK, Kellar C, Morgan C (2018). Tracking active learning in the medical school curriculum: a learning-centered approach. J Med Educ Curric Dev.

[7] Cleghorn GD, Headrick LA (1996). The PDSA cycle at the core of learning in health professions education. Jt Comm J Qual Improv.

[8] Langley GJ, Moen RD, Nolan KM, Nolan TW, Norman CL, Provost LP. The improvement guide: a practical approach to enhancing organizational performance: John Wiley & Sons; 2009.

[9] Standards, Publications, & Notification Forms | LCME [Internet]. [cited 2021 May 14]. Available from: https://lcme.org/publications/

[10] Barzansky B, Hunt D, Moineau G, Ahn D, Lai C-W, Humphrey H, Peterson L (2015). Continuous quality improvement in an accreditation system for undergraduate medical education: benefits and challenges. Med Teach.

[11] Vordenberg SE, Smith MA, Diez HL, Remington TL, Bostwick JR (2018). Using the plan-do-study-act (PDSA) model for continuous quality improvement of an established simulated patient program. Innov Pharm.

[12] Coleman MT, Headrick LA, Langley AE, Thomas JX (1998). Teaching medical faculty how to apply continuous quality improvement to medical education. Jt Comm J Qual Improv.

[13] Silverberg SL, Zannella VE, Countryman D, Ayala AP, Lenton E, Friesen F, Law M (2017). A review of antimicrobial stewardship training in medical education. Int J Med Educ.

[14] Schwartz B. How to Make ID Learning Active. In Idsa; 2016 [cited 2020 Jul 20]. Available from: https://idsa.confex.com/idsa/2016/webprogram/Session7950.html

[15] Gentry SV, Gauthier A, Ehrstrom BL, Wortley D, Lilienthal A, Car LT (2019). Serious gaming and gamification education in health professions: systematic review. J Med Internet Res.

