# Supplementary material for: Applying the Plan-Do-Study-Act cycle in medical education to refine an antibiotics therapy active learning session
Source: BMC Med Educ. 2021 Aug 30;21:459. doi: 10.1186/s12909-021-02886-3 (PMC8404352; doi:10.1186/s12909-021-02886-3)
Supplement: Supplementary file 1 — Additional file 1. Antibiotic Chart. [file 12909_2021_2886_MOESM1_ESM.pdf]

Class	Example(s)	Mechanism of action	Spectrum	Side effects / Notes
**BETA-LACTAMS**				
**PENICILLINS**				
Natural Penicillins	PCN G<br>PCN VK (oral)	all beta lactam antibiotics bind penicillin-binding-proteins (PBP's) to inhibit bacterial cell wall synthesis<br><br>NOTE: antimicrobial effect depends on *time over MIC*	GPC (streptococci, enterococci, PCN-S staph)<br>*N. meningitidis*<br>*T. pallidum* (syphilis)<br>Actinomycosis	Allergy / rash<br>GI intolerance including *C diff*<br>Neurologic (encephalopathy / seizure)<br>immune-mediated cytopenias<br>JH reaction (when treating syphilis)<br>(NOTE: these apply to all beta-lactams - additional / idiosyncratic SE's listed for each class below)
Penicillinase-resistant (anti-staphylococcal) Penicillins	Nafcillin<br>Oxacillin		MSSA	interstitial nephritis (nafcillin)<br>hepatitis (oxacillin)
Aminopenicillins	Amoxicillin (oral)<br>Ampicillin (IV)		similar to natural penicillins but better gram neg coverage (e.g. *H. flu* , *E. coli*)	rash (idiosyncratic with EBV)
Anti-Pseudomonal Penicillins	Ticarcillin<br>Piperacillin		improved gram negative coverage including *Pseudomonas* and anaerobes (e.g. *Bacteriodes fragilis*)	leukopenia and thrombocytopenia including bleeding diathesis (rare)<br>interstitial nephritis<br>hepatitis
**CEPHALOSPORINS**				
General information	see below for each generation	all beta lactam antibiotics bind penicillin-binding-proteins (PBP's) to inhibit bacterial cell wall synthesis<br><br>NOTE: antimicrobial effect depends on *time over MIC*	rule of thumb: successive generations have increasing gram neg and decreasing gram pos coverage; enterococci are instrinsically resistant to cephalosporins	usually well-tolerated even in persons with reported PCN allergy<br><br>SE profile similar to PCN's: allergic reactions (often with eosinophilia), hepatitis, cytopenias, and CNS effects (confusion, seizures)
1st gen	Cephalexin (oral)<br>Cefazolin (IV)		Primarily gram positives e.g. MSSA, streptococci<br>Some aerobic gram neg e.g. *E. coli* , *Klebsiella*<br>Poor anaerobic coverage	
2nd gen	Cefuroxime (oral)<br>Cefotetan<br>Cefoxitin		still some activity vs. gram positives but used more commonly for gram neg aerobes (*H. flu* ; *Moraxella;* *E. Coli*)<br>Cefotetan and Cefoxitin are active against *Bacterioides fragilis* but resistance is increasing	Disulfiram-like reaction with ETOH (cefotetan)
3rd gen	Cefdinir (oral)<br>Cefpoxodime (oral)<br>Cefotaxime<br>Ceftazidime<br>Ceftriaxone		enhanced gram neg coverage due to beta-lactamase stability<br>less activity against gram positives BUT cefotaxime and ceftriaxone remain active vs. *S. pneumo*<br>good CSF penetration<br>Ceftazidime active vs. *Pseudomonas*<br>Common uses: *N. meningitidis; N. gonorrhea; S. pneumo; Enterobacteriaceae*	biliary sludge (ceftriaxone)
4th gen	Cefepime		excellent gram neg including *Pseudomonas;* poor activity vs. gram pos cocci; good CSF penetration	injection site reaction; GI intolerance; cytopenias; hypophosphatemia
5th gen	Ceftaroline		Staph aureus (including MRSA, VRSA, VISA); streptococci; *E. faecalis* ; Enterobacteriaceae (but NOT *Pseudomonas)*	GI intolerance; cytopenias; hypokalemia; pruritis

Class	Example(s)	Mechanism of action	Spectrum	Side effects / Notes
**MONOBACTAMS**	Aztreonam	all beta lactam antibiotics bind penicillin-binding-proteins (PBP's) to inhibit bacterial cell wall synthesis<br><br>NOTE: antimicrobial effect depends on *time over MIC*	gram neg *only*, including *Pseduomonas* (if not resistant); particularly for PCN-allergic patients	safe in patients with PCN allergy
**CARBAPENEMS**	Imipenem-cilastatin<br>Meropenem<br>Ertapenem<br>Doripenem	all beta lactam antibiotics bind penicillin-binding-proteins (PBP's) to inhibit bacterial cell wall synthesis<br><br>NOTE: antimicrobial effect depends on *time over MIC*	gram pos, neg and anaerobes; particularly useful for enterobacteriaceae with extended spectrum beta-lactamase (ESBL); note: ertapenem does NOT have activity vs. Pseudomonas	usually well-tolerated even in persons with reported PCN allergy<br><br>SE profile similar to PCN's: allergic reactions (often with eosinophilia), hepatitis, cytopenias, and seizures (especially with imipenem-cilastatin in setting of renal insufficiency)
**BETA-LACTAM / BETA-LACTAMASE INHIBITOR COMBINATIONS**	see below for each generation	bacteria produce beta-lactamases to break up the beta-lactam ring, thus rendering beta-lactam antibiotics ineffective; by adding a beta-lactamase inhibitor, these drugs restore the beta-lactam activity and extend the antimicrobial spectrum		SE profile similar to other beta-lactams: allergic reactions (often with eosinophilia), hepatitis, interstitial nephritis; cytopenias, and CNS toxicity (seizures) especially in setting of renal failure
	Amoxicillin-clavulanate		MSSA and beta-lactamase producing *H. flu*	rash (idiosyncratic with EBV)
	Ampicillin-sulbactam		MSSA and beta-lactamase producing *H. flu*, anaerobes; *Acinetobacter*	
	Ticarcillin-clavulanate<br>Piperacillin-tazobactam		aerobic gran neg including *Pseudomonas*	high sodium content<br>platelet dysfunction
	Ceftolazone-tazobactam<br>Ceftazidime-avibactam		aerobic gram neg including *Pseudomonas* and most ESBL Enterobacteriaceae (complicated UTI / intra-abdominal infection)	lower cure rates in patients with reduced GFR
	Meropenem-Vaborbactam		aerobic and anaerobic gram positives and negatives, including *Pseudomonas,* ESBL Enterobacteriaceae and some carbapenemase-producing gram negatives (such as KPC); approved for complicated UTI	injection site reaction; GI intolerance; elevation of liver function tests; hypokalemia; lowered seizure threshold

Class	Example(s)	Mechanism of action	Spectrum	Side effects / Notes
**NON-BETA-LACTAM ANTIBIOTICS**				
**QUINOLONES**	Ciprofloxacin<br>Levofloxacin<br>Moxifloxacin	block DNA synthesis (via gyrase inhibition); note 100% bioavailability	spectrum varies by drug:<br>cipro - gram neg, including *Pseudomonas*<br>levofloxacin - gram neg but also streptococci, atypical respiratory pathogens *(Legionella; mycoplasma)*<br>moxifloxacin - gram neg including anaerobes, also streptococci, atypical respiratory pathogens, non-tuberculous mycobacteria	QT interval prolongation<br>tendinopathy / rupture
**AMINOGLYCOSIDES**	Gentamicin<br>Tobramycin<br>Amikacin	inhibit protein synthesis (bind 30S ribosomal subunit); NOTE: *concentration-dependent* killing with post-antibiotic effect	aerobic gram neg bacilli, including *Pseudomonas;* atypical mycobacteria; part of combination therapy for some forms of endocarditis (e.g. enterococcus, certain streptococci, prosthetic valve endocarditis)	nephrotoxicity<br>ototoxicity (can be irreversible)
**TETRACYCLINES**	Tetracycline<br>Doxycycline	inhibit protein synthesis (bind 30S ribosomal subunit)	atypical respiratory pathogens (*Chlamydia* , *Mycoplasma*)<br>STI's (1st line for *Chlamydia;* also gonorrhea and syphilis in PCN-allergic patients<br>multiple vector-born diseases e.g. RMSF and other rickettsial infections, Lyme disease	GI upset<br>esophageal ulcerations (pill esophagitis)<br>photosensitivity<br>stains / deforms teeth in children
**GLYCYLCYCLINE**	Tigecycline	inhibit protein synthesis (bind 30S ribosomal subunit)	broad spectrum - MRSA, VRE, ESBL gram negatives, *C diff*<br>however, side effects and poor blood levels limit utility (cannot use in bacteremia)	nausea / vomiting<br>renal and liver toxicity<br>some reports of increased mortality with use
**LINCOSAMIDES**	Clindamycin	inhibit protein synthesis (bind 50S ribosomal subunit)	gram positive aerobes (strep, staph including MRSA); anaerobes "above the belt" (*Fusobacterium* ; *Prevotella* ; *Peptostreptococcus*)	most likely to cause *C diff*
**MACROLIDES**	Erythromycin (rarely used)<br>Azithromycin<br>Clarithromycin	inhibit protein synthesis (bind 50S ribosomal subunit)	most upper respiratory pathogens (*S. pneumo* , *moraxella* , *H. flu*) and atypical PNA pathogens (*Legionella* , *Chlamydia* , *Mycoplasma*); some gram negative rods such as causes of traveler's diarrhea (*E. coli* , *Shigella*); STI's (*N. gonorrhea* ; *Chlamydia*); part of treatment for atypical mycobacteria (e.g. MAC)	GI intolerance<br>QT prolongation (one study with increased risk of CV death)
**NITROIMIDAZOLES**	Metronidazole	disrupts host cell DNA	anaerobes "below the belt" (bacterial vaginosis; *Bacteroides* ; *Clostridium* including *C diff*); *Trichomonas*	GI intolerance (common)<br>Disulfiram-like reaction with ETOH<br>CNS effects (neuropathy, encephalopathy, tremors)
**OXAZOLIDINONES**	Linezolid<br>Tedizolid	inhibit protein synthesis (bind 50S ribosomal subunit)	gram positives, including MRSA, MSSA, strep, enterococcus including VRE	bone marrow suppression (especially anemia and thrombocytopenia)<br>serotonin syndrome (if given with SSRI)<br>lactic acidosis<br>optic neuritis or peripheral neuropathy

Class	Example(s)	Mechanism of action	Spectrum	Side effects / Notes
GLYCOPEPTIDE	Vancomycin	inhibits bacterial cell wall synthesis by binding D-ala-D-ala	gram positives, including MRSA, vanc-S enterococcus; *C diff* (with oral administration)<br>NOTE - inferior to anti-staph PCN for treatment of MSSA	Red man syndrome (note - NOT an allergy; improves with prolonged infusion)<br>phlebitis<br>renal dysfunction<br>allergic reaction with rash<br>neutropenia, thrombocytopenia
CYCLIC LIPOPEPTIDES	Daptomycin	binds bacterial membrane causing depolarization of membrane potential and cell death	gram positives, including MSSA, MRSA, VRE	elevated CPK and/or myopathy (d/c statins during therapy)<br>elevated LFT's<br>eosinophilic pneumonia
POLYMYXINS	Colistin (Polymyxin E)<br>Polymyxin B	disupts cell membrane	reserved for treatment of resistant gram negatives due to toxicities<br>NOTE: dosing is complex and depends on formulation	nephrotoxicity<br>neurotoxicity (paresthesia, vertigo, visual problems)
RIFAMYCINS	Rifampin<br>Rifabutin	inhibit bacterial DNA-dependent RNA polymerase	tuberculosis and non-tuberculous mycobacteria<br>adjunctive therapy for some forms of staphylococcal endocarditis or infections involving a biofilm (e.g. prosthetic joint infections)<br>should not be used as monotherapy due to rapid emergency of resistance	many drug interactions as revs up P450 system<br>causes red-orange discoloration of body fluids<br>hepatitis
SULFONAMIDES	Trimethoprim-Sulfamethoxazole	inhibits enzymes along bacterial folic acid pathway	broad spectrum including Staph (MRSA and MSSA); gram negatives especially in treatment of UTI; PCP and Toxoplasmosis; *Nocardia* ; *Listeria;* non-tuberculous mycobacteria	rash (including severe reactions e.g. SJS)<br>GI intolerance<br>elevated Cr (due to decreased secretion of Cr)<br>hyperkalemia, possible Type IV RTA<br>bone marrow suppression<br>hemolysis and/or methemoglobinemia in patients with G6PD deficiency<br>hepatitis (often cholestatic)
SULFONES	Dapsone	inhibits enzymes along bacterial folic acid pathway	Leprosy; PCP and Toxoplasmosis	rash (including severe reactions e.g. SJS)<br>hepatitis<br>blood dyscrasias including methemoglobinemia (with or without G6PD deficiency)
NITROFURANTOIN	Nitrofurantoin	bacterial flavoproteins metabolize the drug to reactive intermediates that disrupt ribosomal proteins	UTI pathogens (*E. coli* , *Staph saprophyticus* , *E. faecalis* , some *Enterobacter sp.*)<br>NOTE: drug is inactivated by most body tissues, so only effective in urinary tract	acute hypersensitivity with pulmonary symptoms / infiltrates<br>Pulmonary fibrosis with long-term use<br>hemolysis and methemoglobinemia with G6PD deficiency<br>Neuropathy<br>Lupus-like reaction

Class	Example(s)	Mechanism of action	Spectrum	Side effects / Notes
ANTI-FUNGALS				
POLYENES	Amphotericin B (deoxycholate B; lipid formulations)<br>Nystatin	bind sterols of fungal cell wall leading to leakage of cellular contents and cell death	**Amphotericin:** very broad antifungal spectrum<br>**Nystatin:** limited to oral / topical use for *Candida* infections	**Amphotericin:**<br>"shake and bake" infusion reaction with chills, rigors (seen with older formulation - amphotericin B deoxycholate)<br>nephrotoxicity and electrolyte disurbances (less common with lipid formulations)<br>**Nystatin:** GI / skin irritation
ECHINOCANDINS	Caspofungin<br>Micafungin<br>Anindulafungin	block fungal cell wall synthesis by inhibiting beta-D-glucan synthesis	*Candidas spp.* (including most azole-resistant strains)<br>*Aspergillus*<br>NOTE: not effective against *Cryptococcus sp.* or Mucorales	generally very well tolerated<br>hepatotoxicity but typically asymptomatic elevation of LFT's
AZOLES	Triazoles:<br>Fluconazole<br>Itraconoazole<br>Voriconazole<br>Posaconazole<br>Isavuconazole<br><br>Imidazoles:<br>Ketoconazole	block fungal cell wall synthesis by inhibiting ergosterol synthesis	Vary by drug<br>**Fluconazole:** active against yeasts including *Candida* ; *Cryptococcus* ; NOT effective against molds<br>**Itraconazole:** fairly broad spectrum including treatment of endemic fungi, but use limited by inconsistent bioavailability<br>**Voriconazole:** *Aspergillus* and other resistant molds (*Scedosporium* ; *Fusarium*); fluconazole-resistant *Candida* (*C. glabrata* ; *C. krusei*)<br>**Posaconazole and Isavuconazole**: similar to voriconazole but with addition of activity against Mucorales<br><br>**Ketoconazole:** rarely used due to SE profile / drug interactions	All: hepatotoxicity<br><br>Drug specific:<br>**Fluconazole:** dry skin, alopecia; renal toxicity<br>**Itraconazole:** requires food and acidic gastric pH for absroption (tell patients to drink a Coke); cardiac dysfunction<br>**Voriconazole:** vision changes / hallucinations; photosensitivity; QT prolongation; periostitis (with elevation in serum fluoride levels)<br>**Posaconazole:** QT prolongation<br>**Isavuconazole:** GI intolerance; peripheral edema; *shortens* QT interval<br><br>**Ketoconazole:** adrenal insufficiency
