# Supplementary material for: Applying the Plan-Do-Study-Act cycle in medical education to refine an antibiotics therapy active learning session
Source: BMC Med Educ. 2021 Aug 30;21:459. doi: 10.1186/s12909-021-02886-3 (PMC8404352; doi:10.1186/s12909-021-02886-3)
Supplement: Supplementary file 2 — Additional file 2. Antimicrobial Therapeutics Cases: Facilitator’s Guide. [file 12909_2021_2886_MOESM2_ESM.docx]

**Antimicrobial Therapeutics Cases: Facilitator’s Guide**

**Materials:**

- **Cases**
- **Answer choices for each case**
- **Index cards**

**Instructions:**

**For each case, we will:**

1. **Read the case aloud**
2. **Then we will start the timer**
3. **Each group will write down on index cards the relevant “bugs” and/or “drugs” and will drop the cards in the corresponding paper bags for that case**
4. **Once everyone has turned in their cards, we will go through the answers, starting with the group who turned in their answers FIRST**
5. **The FIRST group to answer the questions CORRECTLY will WIN that round**
6. **The game will be repeated for each case**

**Case 1:**

**23 year-old female with no significant past medical history presents to clinic with a 2-day history of burning with urination, with scant hematuria. She denies fever or flank pain. She also denies any vaginal discharge. She engages in protected sex (condoms) with one male partner approximately twice per week. Her last menstrual period was 10 days ago.**

**Bugs?**

Correct answers:

- *Escherichia coli*
- *Staph saprophyticus*

Other choices:

- Beta-hemolytic Group A Strep
- *Pseudomonas aeruginosa*
- Vancomycin-resistant enterococcus (VRE)

**Drugs?**

Correct answers:

- Nitrofurantoin
- Bactrim

Other choices:

- Vancomycin
- Ciprofloxacin (try to avoid in uncomplicated cystitis)
- Amoxicillin

**References:**

- Gupta K, et al. International Clinical Practice Guidelines for the Treatment of Acute Uncomplicated Cystitis and Pyelonephritis in Women: A 2010 Update by the Infectious Disease Society of America and thte European Society for Microbiology and Infectious Diseases. *Clin Infect Dis* 2011;52(5):e103-e120. <http://www.idsociety.org/Guidelines/Patient_Care/IDSA_Practice_Guidelines/Infections_by_Organ_System/Genitourinary/Uncomplicated_Cystitis_and_Pyelonephritis_(UTI)/>
- <http://www.fda.gov/Drugs/DrugSafety/ucm511530.htm>

**Case 2:**

**A 38 year-old male non-smoker with past medical history significant only for essential hypertension presents to the clinic complaining of cough. His symptoms began 5 days ago with nasal congestion, rhinorrhea and sore throat, but without fever or myalgias. Over the last few days, he has developed a dry, hacking cough, but denies shortness of breath or pleuritic chest pain. On exam, he is afebrile, with no sinus tenderness. Scattered wheezing is audible on pulmonary examination.**

**Bugs?**

Correct answers:

- Respiratory viruses (adenovirus, influenza, respiratory syncytial virus)
- *Mycoplasma pneumoniae –* possible but less likely
- *Chlamydophila pneumoniae –* possible but less likely

Other choices:

- *Mycoplasma genitalium*
- *Staphylococcus aureus*
- *Escherichia coli*

**Drugs?**

Correct answer:

- No antibiotic treatment!

Other choices:

- Vancomycin
- Azithromycin
- Doxycycline
- Moxifloxacin

**References:**

- Albert RH. Diagnosis and Treatment of Acute Bronchitis. *Am Fam Physician.* 2010 Dec 1;82(11):1345-1350. <http://www.aafp.org/afp/2010/1201/p1345.html>
- CDC Get Smart: <https://www.cdc.gov/getsmart/community/for-hcp/outpatient-hcp/adult-treatment-rec.pdf>

**Case 3:**

**A 60 year-old male with a history of coronary artery disease, status post coronary artery bypass grafting (CABG) three months ago, presents with a two-day history of erythema and tenderness of the left lower leg, near the saphenous vein harvesting site. On examination, he has a low grade fever (100.1 degrees F), and the erythema appears to extend up the thigh in a streaking pattern. There is small, tender lymphadenopathy in the left inguinal area as well. Overall, the patient is non-toxic-appearing.**

**Bugs?**

Correct answers:

- Beta-hemolytic Group A Strep – most likely
- Staphylococcus aureus

Other choices:

- *Candida albicans*
- *Klebsiella pnuemoniae*
- *Chlamydia trachomatis*
- *Propionobacterium acnes*

**Drugs?**

Correct answers:

- Penicillin
- Ceftriaxone
- Clindamycin
- +/- Vancomycin
- +/- Linezolid
- +/- Ceftaroline

Other choices:

- Ceftazidime / avibactam
- Meropenem
- Fluconazole[See comment in PubMed Commons below](https://www.ncbi.nlm.nih.gov/pubmed/6982013#comments)

**References:**

- Baddour LM and Bisno AL. Recurrent cellulitis after saphenous venectomy for coronary bypass surgery. [*Ann Intern Med.*](https://www.ncbi.nlm.nih.gov/pubmed/6982013) 1982 Oct;97(4):493-6.
- Stevens DL, et al. Practice Guidelines for the Diagnosis and Management of Skin and Soft Tissue Infections: 2014 Update by the Infectious Diseases Society of America. *Clin Infect Dis* 2014;59(2):e10-52. DOI: 10.1093/cid/ciu296. <http://www.idsociety.org/Templates/Content.aspx?id=32212255985>

**Case 4:**

**A 71 year-old male smoker with a history of Rheumatoid Arthritis, on chronic adalimumab therapy, presents to an urgent care clinic with complaints of fever and cough for 2 days. Cough is productive of yellow-green sputum, with no hemoptysis. He reports easy fatigability and shortness of breath with minimal exertion, with some discomfort with deep inspiration. He denies night sweats, myalgias or sinus tenderness. He reports that he is regularly around his young grandchildren who are “constantly sick.” Chest x-ray shows a patchy infiltrate in the left lingular area. The patient does not meet criteria for hospital admission based on the CURB-65 criteria.**

**Bugs?**

Correct answers:

- *Streptococus pnuemoniae*
- *Legionella pneumophila*
- *Mycoplasma pnuemoniae –* less likely
- *Chlamydophila pnuemoniae* – less likely
- Viral respiratory pathogen(s)

Other choices:

- *Listeria monocytogenes*
- *Histoplasma capsulatum*
- *Mycobacterium tuberculosis*

Note: patients on anti-TNF blockers *are* at risk for tuberculosis, endemic fungi and certain other infections, but the clinical presentation in this case is acute and without systemic symptoms. This is more consistent with community acquired pneumonia.

**Drugs?**

Correct answers:

- Levofloxacin
- Azithromycin
- Doxycycline

Other choices:

- No antibiotic treatment
- Linezolid
- Amoxicillin
- Ciprofloxacin

**References:**

- Capelastegui A, et al. Validation of a prediction rule for the management of community-acquired pnuemonia. *Eur Respir J* 2006; 27: 151–157: <http://citeseerx.ist.psu.edu/viewdoc/download?doi=10.1.1.330.4028&rep=rep1&type=pdf>
- Mandell LA, et al. Infectious Diseases Society of America / American Thoracic Society Consensus Guidelines on the Management of Community-Acquired Pneumonia in Adults. *Clin Infect Dis* (2007) 44 (Supplement_2):S27-S72. DOI: <https://doi.org/10.1086/511159>. <http://www.idsociety.org/Guidelines/Patient_Care/IDSA_Practice_Guidelines/Infections_by_Organ_System/Lower/Upper_Respiratory/Community-Acquired_Pneumonia_(CAP)/>

**Case 5:**

**A 56 year old female with recent diagnosis of acute lymphoblastic leukemia initiates induction chemotherapy (via chest port) with hyper-CVAD (cyclophosphamide, vincristine, doxorubicin and dexamethasone). She is discharged from the hospital on day #7 of the cycle, and is prescribed ciprofloxacin and fluconazole as antimicrobial prophylaxis. The patient contacts her oncologist on day #10 complaining of fever to 102 degrees F, but without localizing symptoms. The oncologist urges the patient to come to the emergency room, where fever is confirmed, with mild tachycardia (HR 100) and borderline blood pressure (89/55), with normal respiratory rate. Diagnostic work up is initiated including basic labs (showing pancytopenia, including an absolute neutrophil count of 100) and chest X-ray (unremarkable). Blood cultures are drawn and the patient is started on empiric antimicrobial therapy.**

**Bugs?**

Correct answers:

- *Pseudomonas aeruginosa*
- Staphylococcus epidermidis
- Viridans streptococci
- ESBL *Escherichia coli*

Other choices:

- *Neisseria meningitidis*
- Cytomegalovirus
- *Cryptococcus neoformans*

**Drugs?**

Correct answers:

- Cefepime
- Vancomycin
- Meropenem
- Piperacillin / tazobactam

Other choices:

- Levofloxacin
- Amoxicillin / clavulanate
- Acyclovir

**Case 5, continued:**

**The patient is started on empiric Vancomycin and cefepime but remains both febrile and persistently neutropenic after 72 hours. Blood cultures remain negative. CT chest is performed, which is interpreted as showing two pulmonary nodules with surrounding ground glass opacity (“halo sign”).**


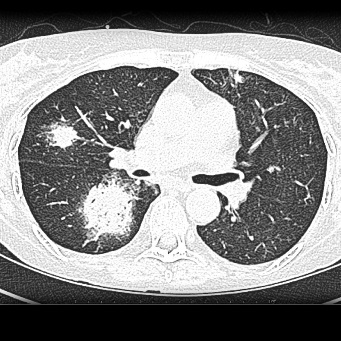


**Case courtesy of A.Prof Frank Gaillard, Radiopaedia.org, rID: 8538**

**Bugs?**

Correct answers:

- *Aspergillus fumigatus*
- *Rhizopus sp.*

Other choices:

- *Staphylococcus aureus*
- *Enterococcus faecalis*
- *Streptococcus pnuemoniae*

**Drugs?**

Correct answers:

- Voriconazole
- Isavuconazole
- Caspofungin
- Liposomal amphotericin B

Other choices:

- Fluconazole
- Itraconazole
- Albendazole

**References:**

- Freifeld AG, et al. Clinical Practice Guideline for the Use of Antimicrobial Agents in Neutropenic Patients with Cancer: 2010 Update by the Infectious Diseases Society of America. *Clin Infect Dis*. 2011;52(4):e56-e93. DOI: 10.1093/cid/cir073: <https://www.idsociety.org/uploadedFiles/IDSA/Guidelines-Patient_Care/PDF_Library/FN.pdf>
- Maertens JA et al. Isavuconazole versus voriconazole for primary treatment of invasive mould disease caused by Aspergillus and other filamentous fungi (SECURE): a phase 3, randomised-controlled, non-inferiority trial. [*Lancet.*](https://www.ncbi.nlm.nih.gov/pubmed/26684607) 2016 Feb 20;387(10020):760-9. doi: 10.1016/S0140-6736(15)01159-9. Epub 2015 Dec 10. http://www.thelancet.com/pdfs/journals/lancet/PIIS0140-6736(15)01159-9.pdf

**Case 6:**

**An 80 year old male with a history of prostate cancer treated with brachytherapy 10 years prior, with subsequent problems with urinary retention and recurrent urinary tract infections, now presents with a 5-day history of dysuria, frequency and hematuria. He is able to answer questions appropriately, but his wife notes that he seems less alert and interactive over the last few days. On examination, he is febrile to 101.1 degrees F, with suprapubic tenderness but no CVA tenderness. Cardiopulmonary examination is unremarkable. Urine and blood cultures are drawn and are pending.**

**Bugs?**

Correct answers:

- ESBL *Escherichia coli*
- *Klebsiella pneumoniae*
- *Enterococcus faecalis*

Other choices:

- Beta-hemolytic group A streptococcus
- *Bacteroides fragilis*

**Drugs?**

Correct answers:

- Meropenem
- Piperacillin / tazobactam
- Ceftazidime / avibactam

Other choices:

- Bactrim
- Nitrofurantoin

**Case 6, continued:**

**Empiric therapy is initiated with ciprofloxacin, but the patient remains febrile. On hospital day #3, the medical student on the team notes a new systolic murmur in the left lower sternal border. Urine and blood cultures are now growing *Enterococcus faecalis*, although sensitivities are pending. Transthoracic echocardiogram is performed and demonstrates a vegetation on the tricuspid valve.**

**Drugs?**

Correct answers:

- Ampicillin plus gentamicin
- Vancomycin plus gentamicin
- Ceftriaxone plus ampicillin

Other choices:

- Linezolid (static)
- Tigecycline

**References:**

- Nicolle LE, ed. AMMI Canada Guidelines Committee. Complicated urinary tract infection in adults. *Can J Infect Dis Med Microbiol*. 2005 Nov-Dec; 16(6): 349-360. <https://www.ncbi.nlm.nih.gov/pmc/articles/PMC2094997/pdf/JIDMM16349.pd>f
- Baddour LM, et al. Infective Endocarditis in Adults: Diagnosis, Antimicrobial Therapy, and Management of Complications: A Scientific Statement for Healthcare Professionals From the American Heart Association. *Circulation*. 2015 Oct 13;132(15):1435-86. <http://circ.ahajournals.org/content/early/2015/09/15/CIR.000000000000029>

**Case 7: A 30 year old male with HIV (diagnosed 10 years ago, not on antiretroviral therapy) presents to the emergency room with a 5 day history of low grade fever and headache. On examination, he is febrile to 100.6 degrees F, but has no focal neurologic deficits and no frank meningismus. Ocular examination reveals photophobia, sluggish extra-ocular movements. Fundoscopic exam is limited due to the photophobia, but after dilation of the pupils, papilledema is observed bilaterally.**

**Bugs?**

Correct answers:

- *Cryptococcus neoformans*
- *Streptococcus pneumoniae*
- *Neisseria meningitides*
- *Listeria monocytogenes*
- *Treponema pallidum*
- *Mycobacterium tuberculosis*

Other choices:

- Mucormycosis
- *Enterococcus faecalis*
- *Pneumocystis jiroveci*

**Drugs?**

Correct answers:

- Vancomycin / ceftriaxone / ampicillin
- Liposomal amphotericin B + flucytosine

Other choices:

- Vancomycin / Cephalexin
- Micafungin + flucytosine
- Vancomycin / rifampin

**References:**

- Thigpen MC, et al. Bacterial meningitis in the United States, 1998-2007. *N Engl J Med*. 2011;364(21):2016.
- Perfect, JR, et al. Management of Cryptococcal Disease: 2010 Update by the Infectious Diseases Society of America. *Clin Infect Dis* (2010) 50 (3): 291-322. DOI: <https://doi.org/10.1086/649858>
